# Thyroid dysfunction in Greece: Results from the national health examination survey EMENO

**DOI:** 10.1371/journal.pone.0264388

**Published:** 2022-03-04

**Authors:** Paraskevi V. Voulgari, Aliki I. Venetsanopoulou, Natasa Kalpourtzi, Magda Gavana, Apostolos Vantarakis, Christos Hadjichristodoulou, Grigoris Chlouverakis, Grigoris Trypsianis, Yannis Alamanos, Giota Touloumi, on behalf of the EMENO study group

**Affiliations:** 1 Department of Internal Medicine, Rheumatology Clinic, Medical School, University of Ioannina, Ioannina, Greece; 2 Dept of Hygiene, Epidemiology, & Medical Statistics, Medical School, National and Kapodistrian University of Athens, Athens, Greece; 3 Dept of Primary Health Care, General Practice and Health Services Research, Medical School of Aristotle University, Thessaloniki, Greece; 4 Public Health, Medical School, University of Patras, Patra, Greece; 5 Dept of Hygiene and Epidemiology, Medical Faculty, University of Thessaly, Larisa, Greece; 6 Laboratory of Biostatistics, School of Medicine, University of Crete, Crete, Greece; 7 Laboratory of Medical Statistics, Medical School, Democritus University of Thrace, Thrace, Greece; 8 Institute of Epidemiology, Preventive Medicine and Public Health, Corfu, Greece; King Faisal Specialist Hospital and Research Center, SAUDI ARABIA

## Abstract

**Background:**

Nationwide data on thyroid disease prevalence in Greece is lacking. Using the national health examination survey EMENO data resources, we aimed to estimate the prevalence of hypothyroidism and hyperthyroidism and associated risk factors in adults living in Greece.

**Methods:**

A random sample of the adults (≥18 years) living in Greece was drawn by multi-stage stratified random sampling based on the 2011 census. During home visits, trained interviewers administered a standardized questionnaire to study participants. All participants answered questions concerning demographic parameters (e.g., age, sex, degree of urbanization, income) and questions concerning smoking habits, alcohol, dietary habits and psychological parameters such as anxiety and thyroid disease. Weighted logistic regression models were fitted to assess factors associated with thyroid disease.

**Results:**

In total, 6006 individuals were recruited in the Greek Health Examination Survey EMENO (response rate 72%) of whom 5981 were eligible for this study. The prevalence of thyroid disease was 9%, where 0.4% was related to hyperthyroidism and 8.6% to hypothyroidism. The prevalence of thyroid disease was higher in women (14.9%) than men (2.7%) (p<0.001). The highest rates of thyroid disease were observed in former iodine-deficient areas. A decrease in the prevalence of thyroidopathies with increasing alcohol consumption was found. Thyroid disease was associated with anxiety in men. Multivariable regression analysis showed that age, geographic area, and smoking were related to thyroid disease.

**Conclusion:**

The prevalence of thyroid disease in Greece is higher in women. Age, habits, and characteristics of geographic areas determine the distribution of thyroidopathies in Greece.

## Background

Thyroid dysfunction is one of the leading endocrine disorders and represents around 30% to 40% of the patients seen in an endocrine practice [1]. The prevalence of overt hypothyroidism in the general population varies between 0.3% and 3.7% in the USA and between 0.2% and 3.5% in Europe, depending on the definition [2–6]. A meta-analysis of studies across nine European countries estimated that the prevalence of undiagnosed hypothyroidism at around 5%: high or low Iodine intake affects the prevalence of hypothyroidism among various populations [6–8]. Hypothyroidism occurs more frequently in women, older people (>65 years), and white individuals. However, data on ethnic differences are scarce [4, 9, 10]. Hypothyroidism is common in autoimmune diseases, while individuals with Down’s syndrome or Turner’s syndrome have an increased risk of the disease. On the other hand, tobacco smoking and moderate alcohol intake are associated with a reduced risk of hypothyroidism [11, 12]. The prevalence of hyperthyroidism is 0.8% in Europe [6] and 1.3% in the USA [3]. Hyperthyroidism increases with age and is more frequent in women. There are limited data for ethnic differences: African Americans and Asians are much more likely to develop Graves’ disease when compared to whites. On the other hand, whites have an increased risk of Hashimoto’s thyroiditis than other ethnic groups [10]. A decrease in hyperthyroidism incidence after introducing universal salt iodization programs has been reported [13]. Until recently, few studies concerning thyroid dysfunction had been carried out in Greece. These were restricted to specific populations such as neonates, patients with Type 1 and Type 2 diabetes mellitus, post-partum women, and patients with thalassemia major [14–17]. A recent study in Greek healthy adults showed that nutrition habits and anxiety correlated with thyroid dysfunction [18]. However, valid, nationwide estimates of the prevalence of thyroidopathies and associated risk factors are needed in Greece. Recently a national health examination survey named National Survey of Morbidity and Risk Factors (EMENO) was carried out in a representative sample of the adults living in Greece [19].

In this study using EMENO data resources, we aimed to estimate the prevalence of thyroidopathies and the Greek adult population’s associated risk factors.

## Methods

### The EMENO survey

Data were derived from the EMENO health examination survey. EMENO is a population-based, cross-sectional survey conducted from May 2013 to June 2016. Details of the study have been described elsewhere [18]. A random sample of the adults (≥18 years) living in Greece was drawn by multi-stage stratified random sampling based on the 2011 census. During home visits, trained interviewers administered a standardized questionnaire to study participants. All participants provided signed informed consent. The study protocol was approved by the Athens University IRB (http://en.uoa.gr/) and the Hellenic Data Protection Authority (www.dpa.gr).

The participants answered questions concerning demographic parameters (e.g., age, sex, degree of urbanization, income, e.t.c.) and questions concerning smoking habits, alcohol consumption, dietary habits, psychological parameters such as symptoms of anxiety and depression (using the Patient Health Questionnaire-4 or PHQ-4)) [20], and other chronic diseases including thyroidopathies. The level of adherence to Mediterranean diet was measured by the Mediterranean diet adherence screening (MEDAS) questionnaire [21].

Individuals were recorded to have thyroidopathy if they reported a medical diagnosis of thyroidopathy. They were also asked if they were using any thyroid drugs. Standard treatment for hypothyroidism involved daily use of the synthetic thyroid hormone levothyroxine and for hyperthyroidism two antithyroid medications propylthiouracil and methimazole. No other alternative hormone preparations were reported. TSH and thyroid hormones were not measured. As blood thyroid hormones measurements were not performed, cases of subclinical thyroid disease were not included. The majority of participants in the EMENO survey were Caucasians (87,4%), while all individuals who reported having thyroid disease cohort were Caucasians. There were immigrants in the survey, but it was a prerequisite for participating in the study to speak Greek.

### Statistical analysis

Sampling weights, being the reciprocal of the selection probabilities, were applied to adjust for the sampling design; sampling weights were multiplied with post-stratification factors to match the age, gender, and geographical distribution of the sample to that of the Greek population based on the 2011 census provided by the Hellenic Statistical Authority. Weighted medians and interquartile range for continuous variables and weighted percentages for categorical variables were provided. Weighted multivariable logistic regression models were fitted to assess factors associated with thyroidopathies.

## Results

In total, 6006 individuals were recruited in the Greek Health Examination Survey EMENO (overall response rate 72%). Of these, 20 participants were excluded because of missing information. Three participants who reported a recent thyroidectomy history with no further information were not included in the study. There were no cases of gestational transient thyrotoxicosis or participants that had recently received radioactive iodine treatment. Another patient who was currently scheduled for thyroidectomy with no further details was excluded from the cohort. At last, one patient with a history of subacute thyroiditis and no data regarding the thyroid profile was not included in the analysis.

Finally, five thousand nine hundred and eighty-one individuals were included in the study: 51.4% females with a median (interquartile range (IQR)) age 49 (35–65.6) years and 48.6% males with a median age 46.6 (33.1–62) years. The overall prevalence of thyroid disease was 9.0% (8.2, 9.8), where 0.4% was related to hyperthyroidism and 8.6% to hypothyroidism. The prevalence of thyroid disease differed significantly between men and women (2.7% in men, 14.9% in women, p-value <0.001); 14.4% of women had hypothyroidism, and 0.5% hyperthyroidism. Tables 1 and 2 show the prevalence of overall thyroid disease, and hypothyroidism, respectively, according to age, sex, region, and eating habits. Prevalence of overall hyperthyroidism according to age, sex, region and eating habits is presented in S1 Table.

**Table 1:** Prevalence of thyroid disease overall and by gender.

	Total (N = 5981)	Female (N = 3437)	Male (N = 2544)
**Age group (years)**	n/N (weighted %)	n/N (weighted %)	n/N (weighted %)
18–29	24/633 (3.7)	16/335 (4.7)	8/298 (2.7)
30–39	54/819 (6)	49/469 (10.7)	5/350 (1.4)
40–49	115/1018 (10.3)	104/612 (17.6)	11/406 (2.8)
50–59	155/1111 (12.3)	146/686 (21.5)	9/425 (2.7)
60–69	160/1109 (14)	141/626 (23.2)	19/483 (4.1)
70+	116/1291 (9.6)	98/709 (14.4)	18/582 (3.1)
**p-value**	<0.001	<0.001	0.432
**Region**			
Athens	196/1645 (10.2)	171/955 (16.1)	25/690 (3.6)
Crete	61/394 (13.2)	55/214 (23.1)	6/180 (3)
Thessaloniki	57/579 (8)	52/344 (13.5)	5/235 (1.8)
Thrace	23/318 (5.9)	19/175 (9.9)	4/143 (1.7)
Thessaly	31/475 (4.6)	29/289 (8.2)	2/186 (1)
Peloponnese	49/582 (7.3)	43/324 (12.4)	6/258 (2.3)
Epirus	25/307 (6.8)	21/178 (10.5)	4/129 (2.9)
Corfu	14/250 (4.1)	14/145 (8.1)	0/105 (0)
Central Greece	28/403 (6.3)	25/215 (11.4)	3/188 (1.2)
Macedonia	98/665 (11.9)	88/389 (20.1)	10/276 (3.4)
Lesvos-Rhodes	42/363 (9)	37/209 (15.1)	5/154 (3)
**p-value**	<0.001	<0.001	0.355
**Degree of urbanization**			
urban	368/3417 (9.4)	328/2042 (15)	40/1375 (3)
semi-urban	115/1060 (9.2)	101/596 (15.5)	14/464 (2.7)
rural	141/1504 (7.7)	125/799 (14.2)	16/705 (1.9)
**p-value**	0.180	0.805	0.325
**Smoking status**			
Ever smoker	258/3055 (7.1)	205/1296 (14.0)	53/1759 (2.8)
Never smoker	361/2794 (11.5)	344/2073 (15.9)	17/721 (2.7)
**p-value**	<0.001	0.126	0.909
**alcohol consumption** (drinks\last week)			
0–2	515/3894 (11.7)	477/2784 (16.0)	38/1110 (3.5)
3+	97/1867 (4.4)	66/533 (10.7)	31/1314 (2.2)
**p-value**	<0.001	0.003	0.075
**Red meat consumption** (daily servings)			
≥ 1	140/1662 (7.2)	119/851 (13.2)	21/811 (2.4)
<1	479/4232 (9.9)	430/2540 (15.7)	49/1692 (2.9)
**p-value**	0.001	0.082	0.517
**Fruit consumption** (daily servings)			
<3	511/5111 (8.8)	451/2923 (14.5)	60/2188 (2.8)
≥3	107/776 (10.8)	97/460 (18.8)	10/316 (2.2)
**p-value**	0.065	0.018	0.508
**Vegetable consumption** (daily servings)			
<1	178/1946 (7.8)	156/993 (14.9)	22/953 (2.3)
≥2 of cooked or 1 serving of raw vegetables / salad	442/3949 (9.7)	394/2398 (15.1)	48/1551 (3)
**p-value**	0.020	0.867	0.349
**Symptoms of Stress**			
No	431/4354 (8.5)	385/2352 (15.2)	46/2002 (2.3)
Yes	178/1450 (10.9)	154/981 (14.5)	24/469 (5.1)
**p-value**	0.008	0.662	0.002
**Symptoms of Depression**			
No	459/4735 (8.2)	404/2598 (14.2)	55/2137 (2.5)
Yes	148/1070 (12.8)	134/735 (17.7)	14/335 (4.5)
**p-value**	<0.001	0.025	0.062

**Table 2:** Prevalence of hypothyroidism (overall and by gender).

	Total	Female	male
	n/N (weighted %)	n/N (weighted %)	n/N (weighted %)
**Age group**			
18–29	24/633 (3.7)	16/335 (4.7)	8/298 (2.7)
30–39	51/819 (5.7)	46/469 (10.2)	5/350 (1.4)
40–49	112/1018 (10)	101/612 (17.1)	11/406 (2.8)
50–59	147/1111 (11.9)	139/686 (20.7)	8/425 (2.6)
60–69	153/1109 (13.4)	136/626 (22.2)	17/483 (3.7)
70+	109/1291 (8.9)	94/709 (13.8)	15/582 (2.5)
**p-value**	<0.001	<0.001	0.560
**Region**			
Athens	191/1645 (9.9)	167/955 (15.8)	24/690 (3.5)
Crete	59/394 (12.9)	53/214 (22.4)	6/180 (3)
Thessaloniki	54/579 (7.5)	49/344 (12.6)	5/235 (1.8)
Thrace	21/318 (5.5)	19/175 (9.9)	2/143 (.9)
Thessaly	30/475 (4.5)	28/289 (7.9)	2/186 (1)
Peloponnese	47/582 (7)	42/324 (12)	5/258 (2.1)
Epirus	23/307 (6.3)	19/178 (9.5)	4 /129(2.9)
Corfu	13/250 (3.7)	13/145 (7.2)	0/105 (0)
Central Greece	26/403 (5.9)	24/215 (11)	2/188 (.70)
Macedonia	93/665 (11.3)	84/389 (19.2)	9/276 (3.1)
Lesvos-Rhodes	39/363 (8.3)	34/209 (13.7)	5/154 (3)
**p-value**	<0.001	<0.001	0.246
**Degree of urbanization**			
urban	355/3417 (9.1)	316/2042 (14.5)	39/1375 (3)
suburban	108/1060 (8.6)	94/596 (14.4)	14/464 (2.7)
rural	133/1504 (7.2)	122/799 (13.9)	11/705 (1.3)
**p-value**	0.130	0.930	0.072
**Smoking status**			
Ever smoker	241/3055 (6.7)	194/1296 (13.3)	47/1759 (2.6)
Never smoker	350/2794 (11.2)	333/2073 (15.4)	17/721 (2.7)
**p-value**	<0.001	0.098	0.853
**Alcohol consumption** (drinks/last week)			
0–2	491/3894 (11.2)	457/2784 (15.4)	34/1110 (3.2)
3+	93/1867 (4.2)	64/553 (10.4)	29/1314 (2.1))
**p-value**	<0.001	0.005	0.112
**Red meat (daily amount)**			
> = 1 servings	137/1662 (7)	117/851 (12.9)	20/811 (2.3)
<1 serving	454/4232 (9.5)	410/2540 (15)	44/1692 (2.7)
**p-value**	**0.004**	**0.151**	**0.620**
**Fruit consumption (daily amount)**			
<3	488/5111 (8.4)	433/2923 (13.9)	55/2188 (2.7)
> = 3	102/776 (10.3)	93/460 (18.2)	9/316 (1.9)
**p-value**	0.079	0.020	0.332
**Vegetable consumption (daily amount)**			
<1 serving	173/1946 (7.6)	152/993 (14.5)	21/953 (2.3)
> = 2 of cooked or 1 serving of raw vegetables / salad daily	419/3949 (9.2)	376/2398 (14.5)	43/1551 (2.8)
**p-value**	0.042	0.998	0.479
**Symptoms of Stress**			
No	408/4354 (8.1)	367/2352 (14.5)	41/2002 (2.1)
Yes	174/1450 (10.8)	151/981 (14.3)	23/469 (5)
**p-value**	0.003	0.894	0.001
**Symptoms of Depression**			
No	438/4735 (7.9)	387/2598 (13.7)	51/2137 (2.4)
Yes	142/1070 (12.3)	130/735 (17.3)	12/335 (4.1)
**p-value**	<0.001	0.021	0.112

### Univariable analysis

In women, rates of thyroid disease tended to increase by age up to 60–69, whereas there was a slight decrease in the prevalence in those over the age of 70s. In men, on the other hand, there was no significant difference with age. The highest rates of thyroid disease were observed in Crete, Macedonia (excluding Thessaloniki), and Athens, while the lowest rates were observed in Thessaly and Corfu for both men and women. Regarding the degree of urbanization, only in men, there was a tendency for higher prevalence of thyroid disease as higher the degree of urbanization, but the differences were not statistically significant.

Investigating the participants’ eating habits, women who ate <1 serving of red meat per week and ≥ 3 fruits per day appeared to have a higher thyroid disease rate (compared to those consuming> 1 serving of red meat and < 3 fruits per day, respectively), while there was no association between thyroid disease and vegetable consumption. There does not seem to be a difference in the percentages in the specific nutritional factors in men.

Regarding the relationship between thyroid disease and smoking habits overall, there was a significant association with higher prevalence among never smokers. When stratifying though by gender there were no significant associations’, indicating that gender acts as a confounder. Higher alcohol consumption was associated with lower prevalence of thyroid disease overall as well as in both women and men.

In men, thyroid disease seemed to be associated with symptoms of anxiety and of depression, while there was no association between a symptom of anxiety and thyroid disease in women (Table 1).

The number of hyperthyroid participants was too small to find statistically significant associations with the factors we investigated after stratifying by gender (S1 Table). For hypothyroidism, though, as the vast majority of those with thyroid disease suffered from hypothyroidism, gender results were similar to those reported above for overall thyroidopathy (Table 2).

### Multivariable analysis

To assess which of the factors found to be univariably associated with thyroid disease was an independent risk factor, we run multivariable logistic models. Due to the different patterns in women and men, two different models, were fitted, one for each gender. Results from the multivariable logistic regression model fitted to women showed that age, geographic area, alcohol consumption and whether they were ever smokers remained statistically significant factors (Table 3).

**Table 3:** Results from weighted logistic regression analysis to assess the factors independently related to thyroid disease in women.

Covariate	Odds Ratio	95% Conf. Interval	p-value
**Age group**			
18–29*	1		
30–39	2.340	(1.238,4.421)	0.009
40–49	4.222	(2.385,7.474)	<0.001
50–59	5.316	(3.086,9.159)	<0.001
60–69	5.665	(3.214,9.987)	<0.001
70+	2.949	(1.672,5.200)	<0.001
**Region**			
Athens*	1		
Crete	1.692	(1.178,2.430)	0.004
Thessaloniki	0.834	(0.557,1.248)	0.377
Thrace	0.549	(0.322,0.935)	0.027
Thessaly	0.426	(0.261,0.695)	0.001
Peloponnese	0.740	(0.492,1.114)	0.149
Epirus	0.593	(0.355,0.991)	0.046
Corfu	0.472	(0.262,0.850)	0.012
Central Greece	0.657	(0.437,0.988)	0.044
Macedonia	1.285	(0.935,1.768)	0.122
Lesvos-Rhodes	0.980	(0.654,1.467)	0.921
**Ever smoker**			
No*	1		
Yes	0.826	(0.669,1.020)	0.076
**Alcohol consumption** (drinks/last week)			
0–2	1		
3+	0.720	(0.527,0.983)	0.038
* Baseline category			

As in univariable analysis, the odds of having thyroid disease increased with age until the age of 69, while after that, there was a decline, although it remained higher compared to women 18–29 years age group. Regarding the geographical distribution, women living in Crete had increased by 69% odds of having thyroid disease compared to those living in Athens. At the same time there did not seem to be a statistically significant difference between Athens and Thessaloniki, Peloponnese, Macedonia, and Lesvos-Rhodes. Women living in Thrace, Thessaly, Corfu, and Central Greece were less likely to have thyroid disease than those living in Athens, regardless of age, alcohol consumption and smoking habits. Finally, women who were smokers or ex-smokers had decreased odds of having thyroid disease by 17% compared with never-smokers whereas those consuming 3 or more alcohol during last week had decreased odds of having thyroid disease by 28% compared to those consuming 0–2 drinks after adjusting for the rest factors in the model.

In men (Table 4), age was not associated with thyroid disease (p-value = 0.408). On the contrary, there seemed to be a negative correlation between alcohol consumption and thyroid disease, while stress symptoms were associated with higher odds of having thyroid disease (adjusted OR = 2.38, p-value = 0.002).

**Table 4:** Results from weighted logistic regression analysis to assess the factors independently related to thyroid disease in men.

Covariate	Odds Ratio	95% Conf. Interval	p-value
Age, years	1.006	(0.991,1.021)	0.408
Alcohol consumption (drinks/last week)			
0–2*	1		
3+	0.651	(0.393, 1.079)	0.096
Stress			
No*	1		
Yes	2.383	(1.361, 4.173)	0.002
*Baseline category			

## Discussion

In the present study, we estimated the prevalence of thyroidopathies in the Greek adult population and associating risk factors using EMENO data resources. The prevalence of thyroid diseases was 9%, which is higher than in other European countries [5, 6]. Hyperthyroidism was low (0.4%), in accordance with the results of a European meta-analysis [6], while the majority of patients with thyroid dysfunction had hypothyroidism (8.6%). However, there are differences among epidemiologic studies due to various selection criteria of the studied population, the definition and severity of thyroid diseases (e.g., overt and subclinical dysfunction) and the influence of age, sex and environmental factors [6]. Genetic factors may also have influenced differences observed among countries [22, 23]. The prevalence of thyroid diseases, especially hypothyroidism, was higher in women (14.9%) than men (2.7%) (p<0.001). It is known that hypothyroidism occurs more frequently in women [4, 24]. It has been reported that sex steroids have a pathogenetic role in autoimmune thyroid disorders (ATD), a major cause of hypothyroidism [25]. Some thyroid self-antigens are located on the X-chromosome. Thus, it has been hypothesized that the random X chromosome inactivation may influence the incidence of ATD in female and male sex [26]. An increase in thyroid disease rates was observed in older women, while in men, no significant difference with age was found. Many studies have shown increased thyroid disease, especially hypothyroidism, in older people [23, 24]. A major factor responsible for this observation seems to be a gradual thyroid failure in many people caused by autoimmunity [23]. Analysis of the geographical distribution of thyroid disease showed that the highest rates were observed in former iodine deficient-areas. Many parts of Greece throughout the country, mainly mountainous but not in a continuous area, had been characterized in the late 60s as iodine deficient areas: especially those in the western side of Greece and Thessaly [27, 28]. Since then, the situation has improved with more recent data from Greek populations showing a median urinary iodine concentration indicative of borderline iodine deficiency, or even sufficient iodine intake [29]. These observations are probably related to the improvement of socioeconomic conditions and the increased use of iodized salt. Iodine interferes with thyroid function and thyroid diseases [23]. It is known that low iodine intake is associated with signs of insufficient thyroid hormone production and frequent occurrence of goiter and hypothyroidism in older people [23, 30–32]. Investigating the participants’ eating habits, women who ate <1 serving of red meat per week and ≥3 fruits per day appeared to have higher thyroid disease, while there were no differences in men’s specific nutritional factors. However, the results from the multivariable regression analysis did not show that the eating habits were independently related to thyroid disease. The above univariable differences in women’s nutritional consumption may need further investigation in relation to other factors such as body weight, iodine intake and certain eating habits.

A decrease in the prevalence of thyroid disease with increasing alcohol consumption was found in both men and women. Alcohol has been reported to cause direct suppression of thyroid function by cellular toxicity, and indirect suppression by blunting thyrotropin-releasing hormone response [33]. However, most of the results on human studies derive from detoxification programs for alcoholics and thus do not involve social drinkers. Still, regular alcohol consumption has been associated with less prevalence of goiter [23, 34], and overt autoimmune hypothyroidism irrespective of sex and alcohol consumption [10].

Ever smokers had less thyroidopathy in our study, significantly so for women even in the multivariable analysis, but numbers for men were small. The association between tobacco smoking and thyroid function is incompletely understood. Studies so far have shown that it influences the thyroid gland as some of its constituents inhibit iodine uptake and incorporation of iodine into the hormone molecule in the gland. Also, the stimulation of the sympathetic nervous system by cigarette smoke is thought to affect thyroid function [35]. Smoking is negatively associated with hypothyroidism but positively associated with hyperthyroidism [12, 36], a tendency also seen in our study, although without reaching statistical significance, but numbers particularly for men and for hypothyroidism in both sexes were small. It has been reported that the effects of smoking were pronounced in iodine-deficient areas, where smokers present a higher frequency of goiter and increased serum thyroglobulin levels [23]. Smoking may worsen iodine deficiencies by thiocyanate inhibition of the sodium-iodine-symporter [37]. Finally, in the present study, symptoms of anxiety and depression were univariably associated with thyroid disease in men but only symptoms of depression in women. In multivariable analysis, anxiety negative effect persisted in men. Emotional or psychological stress may correlate with ATD because of cortisol’s effects on the immune system [25, 38].

### Strengths and limitations

Our study has a number of strengths. To our knowledge, it is the first study in our country with nationwide estimates of the prevalence of thyroidopathies and associated risk factors. Case definition was based on self-report with a simultaneous record of ongoing treatments. Interviewers questioned participants thoroughly for their medical diagnoses and simultaneously record their medications, requesting clarifications on the diagnoses reported and thus providing a detailed documentation of thyroid disease cases. Thus, there was ensured that cases of levothyroxine treatment used for treating large benign goiters [39] and inappropriately weight loss [40], were not included.

### Our study has some limitations

The collected data concerning hyperthyroidism and hypothyroidism were based on self-reporting and linking to receiving drugs TSH and thyroid hormones were not measured. These measurements are crucial for detecting subclinical thyroid disease and, along with imaging assessment, essential for differentiating causes of hyperthyroidism and thyrotoxicosis [41]. Thus, in our study both hypothyroidism and hyperthyroidism may be underestimated. The exact etiology of hypo- and hyperthyroidism was not recorded. Finally, the sample size was not large enough to make sufficient comparisons for the characteristics of the study population. Thus, in some factors, statistically significant differences could not be detected due to sampling limitations.

In conclusion, we described the prevalence of thyroidopathies in a representative sample of adults living in Greece, and we evaluated the associated risk factors. The prevalence of thyroidopathies was relatively high in women. Age, geographic area, alcohol consumption and smoking were related to thyroid diseases in women whereas alcohol consumption and symptoms of anxiety in men.

## Supporting information

S1 Table Prevalence of hyperthyroidism (overall and by gender). (PDF)
