# Supplementary material for: Thyroid dysfunction in Greece: Results from the national health examination survey EMENO
Source: PLoS One. 2022 Mar 4;17(3):e0264388. doi: 10.1371/journal.pone.0264388 (PMC8896672; doi:10.1371/journal.pone.0264388)
Supplement: S1 Table — (PDF) [file pone.0264388.s001.pdf]

S1 table. Prevalence of hyperthyroidism (overall and by gender).

	**Total**	**Female**	**male**
	n/N (weighted %)	n/N (weighted %)	n/N (weighted %)
**Age group**			
18-29	0/633 (0)	0/335 (0)	0/298 (0)
30-39	3/819 (.3)	3/469 (.5)	0/350 (0)
40-49	3/1018 (.3)	3/612 (.5)	0/406 (0)
50-59	8/1111 (.5)	7/686 (.8)	1/425 (.1)
60-69	7/1109 (.7)	5/626 (1)	2/483 (.4)
70+	7/1291 (.6)	4/709 (.6)	3/582 (.6)
**p-value**	0.075	0.380	0.147
**Region**			
Athens	5/1645 (.2)	4/955 (.3)	1/690 (.1)
Crete	2/394 (.4)	2/214 (.7)	0/180 (0)
Thessaloniki	3/579 (.5)	3/344 (.9)	0/235 (0)
Thrace	2/318 (.4)	0/175 (0)	2/143 (.9)
Thessaly	1/475 (.1)	1/289 (.2)	0/186 (0)
Peloponnese	2/582 (.3)	1/324 (.4)	1/258 (.2)
Epirus	2/307 (.5)	2/178 (1)	0/129 (0)
Corfu	1/250 (.4)	1/145 (.8)	0/105 (0)
Central Greece	2/403 (.4)	1/215 (.4)	1/188 (.5)
Macedonia	5/665 (.6)	4/389 (.9)	1/276 (.4)
Lesvos-Rhodes	3/363 (.7)	3/209 (1.4)	0/154 (0)
**p-value**	0.742	0.589	0.615
**Degree of urbanization**			
urban	13/3417 (.3)	12/2042 (.5)	1/1375 (.1)
semi-urban	7/1060 (.5)	7/596 (1.1)	0/464 (0)
rural	8/1504 (.5)	3/799 (.3)	5/705 (.6)
**p-value**	0.331	0.109	0.008
Smoking status			
Ever smoker	17/3055 (0.4)	11/1296 (0.7)	6/1759 (.2)
Never smoker	11/2794 (0.3)	11/2073 (.5)	0/721 (0)
**p-value**	0.642	0.517	0.126
**alcohol consumption** (drinks/last week)			
0 - 2	24/3894 (0.5)	20/2784 (0.6)	4/1110 (0.3)
3+	4/1867 (0.1)	2/553 (0.3)	2/1314 (0.1)
**p-value**	0.015	0.236	0.274
**Red meat consumption** (daily servings)			

>= 1	3/1662 (.2)	2/851 (.2)	1/811 (.1)
<1	25/4232 (.5)	20/2540 (.7)	5/1692 (.2)
**P-value**	0.058	0.136	0.440
**Fruit consumption** (daily servings)			
<3	23/5111 (.3)	18/2923 (.5)	5/2188 (.1)
≥3	5/776 (.5)	4/460 (.6)	1/316 (.4)
**P-value**	0.491	0.793	0.379
**Vegetable consumption** (daily servings)			
<1	5/1946 (.2)	4/993 (.4)	1/953 (.1)
≥2 of cooked or 1 serving of raw vegetables / salad daily	23/3949 (.5)	18/2398 (.6)	5/1551 (.2)
**p-value**	0.130	0.442	0.178
Symptoms of Stress			
No	23/4354 (.4)	18/2352 (.7)	5/2002 (.2)
Yes	4/1450 (.2)	3/981 (.2)	1/469 (.1)
**p-value**	0.082	0.067	0.395
Symptoms of Depression			
No	21/4735 (.3)	17/2598 (.6)	4/2137 (.1)
yes	6/1070 (.5)	4/735 (.5)	2/335 (.5)
**p-value**	0.483	0.841	0.146
